# The Psychological Impact Among Syrian Refugees in Host Countries

**DOI:** 10.3390/healthcare13050488

**Published:** 2025-02-24

**Authors:** Dalia Al-Hourani, Mahmoud Al-Wriekat, Rocío Llamas-Ramos, Inés Llamas-Ramos

**Affiliations:** 1 Ph.D. Program in Health, Disability, Dependency, and Wellbeing, Faculty of Nursing and Physiotherapy, Universidad de Salamanca, 37007 Salamanca, Spain; daliaalhourani@gmail.com; 2 Clinical Psychology, Faculty of Arts and Sciences, Al-Ahliyya Amman University, Amman 19111, Jordan; mhmwriekat7@gmail.com; 3 Department of Nursing and Physiotherapy, Universidad de Salamanca, Avd. Donantes de Sangre s/n, 37007 Salamanca, Spain; inesllamas@usal.es; 4 Instituto de Investigación Biomédica de Salamanca (IBSAL), Paseo San Vicente, 182, 37007 Salamanca, Spain; 5 University Hospital of Salamanca, 37007 Salamanca, Spain

**Keywords:** mental health, anxiety, depression, stress, Jordan, Spain

## Abstract

**Background**: Refugees face numerous challenges, including conflict, displacement, family separation, human rights violations, limited access to basic needs, and exposure to violence and torture. These experiences significantly impact their psychological well-being, leading to increased stress, anxiety, and depression. Syrian refugees in Jordan may experience higher levels of depression and anxiety than those in Spain due to harsher living conditions, limited economic opportunities, uncertain legal status, inadequate mental health services, and ongoing proximity to the Syrian conflict, whereas refugees in Spain benefit from better integration, stronger support systems, and greater stability. **Objective**: This study aimed to assess and compare the psychological symptoms experienced by Syrian refugees in Jordan and Spain. **Methods**: A cross-sectional correlational design was employed to evaluate the psychological impact on Syrian refugees in both developed (Spain) and developing (Jordan) country. A sample of 200 refugees from camps, residences, and refugee centers was recruited. Psychological symptoms were measured using the Depression Anxiety Stress Scale (DASS-21). **Results**: The majority of participants had low income, secondary education, and were unemployed. Overall, 55.5% reported stress, 63.5% anxiety, and 68.5% depression. Higher levels of stress and depression were observed among females, unemployed individuals, and low-income groups. Age also played a critical role, with individuals aged 40–49 reporting elevated stress, anxiety, and depression compared with other age groups. Discrimination and living arrangements further influenced mental health, with low-social-connectivity communities and rural areas associated with higher anxiety and depression. Higher levels of stress and depression were observed among females in Spain and significant mental health disparities across age and income groups in Jordan. These findings underscore the need for targeted interventions addressing sociodemographic vulnerabilities to improve refugee mental health outcomes. Refugees in Jordan reported significantly higher levels of depression and anxiety compared with those in Spain. **Conclusions**: The findings highlight the profound psychological burden faced by Syrian refugees, particularly in Jordan, where living conditions and systemic challenges exacerbate mental health issues. Factors such as age, gender, marital status, low education, and low income were strongly associated with higher stress levels in both countries. These results underscore the need for targeted mental health interventions, improved access to psychological support, and policies that address the socioeconomic and legal challenges faced by refugees. Enhancing integration, reducing discrimination, and providing comprehensive mental health services are critical steps toward improving the well-being of this vulnerable population.

## 1. Introduction

Forced displacement remains a pressing global issue, with millions fleeing their home countries due to conflict, persecution, and human rights violations. In this study, the term “refugee” is defined according to the 1951 Geneva Convention Relating to the Status of Refugees and its 1967 Protocol. As per these documents, a refugee is an individual who, “*Due to a legitimate fear of persecution based on factors such as race, religion, nationality, membership in a specific social group, or political beliefs, an individual has fled their country of origin and cannot or, because of this fear, chooses not to seek protection from their home country*” [1,2]. The end of World War II in 1945 did not mark the end of human suffering. Tragedies such as genocides, armed conflicts, wars, persecutions, climate change, and ethnicity cleansing persist to this day. By the end of 2022, over 108.4 million people were forced to flee their homes as internally displaced persons, refugees, and asylum seekers [3]. Among these crises, the Syrian conflict stands out as the largest displacement crisis since World War II, with more than 5,019,359 people compelled to leave Syria since 2011 [4]. These staggering figures underscore the ongoing global challenges of displacement and human suffering.

The contexts of Spain and Jordan differ significantly in terms of refugee numbers, living conditions, and integration processes. Spain hosts a smaller number of Syrian refugees, who are integrated into urban areas with support from NGOs and government programs [3,5]. In contrast, Jordan hosts over 1.3 million Syrian refugees, many of whom reside in large refugee camps such as Zaatari and Azraq, facing significant challenges in accessing employment, education, and healthcare [3,6]. These differences highlight the importance of considering host country contexts when examining refugee mental health and integration outcomes [7,8].

Refugees are particularly vulnerable to mental illnesses due to exposure to numerous traumatic and distressing events, including war, perilous journeys from their homelands, sexual violence, and the risks associated with living in detention centers or camps [9]. As a result, refugees often experience significant mental health challenges, such as heightened risks of suicide, anxiety, and depression [10]. Syrian refugees in particular have faced profound mental health and psychosocial impacts due to the stressors of forced displacement, ongoing conflict-related violence, and the challenges of resettlement [11].

The severity of physical, and mental symptoms, compounded by difficulties of living in isolation, significantly impairs refugees’ functioning and well-being. Chronic pain and physical health problems are prevalent among refugees [12,13], with studies showing that chronic pain is common among multi-traumatized refugees in outpatient mental health clinics. The intensity of pain is strongly correlated with the severity of psychiatric symptoms and morbidity [14].

Recent data from the United States and Europe highlight the persistent mental health challenges faced by refugees in post-settlement environments. For example, newly arrived Syrian refugees in Detroit reported depression and anxiety rates of 47.7% and 40.3%, respectively, with women twice as likely to exhibit distress [15]. Similarly, a study in Sweden found that 40.2% of Syrian refugee adults met the criteria for depression, 37.7% reported low subjective well-being, and 31.8% experienced anxiety [16]. Key factors contributing to distress included exposure to interpersonal violence and resettlement stressors such as family conflicts, economic hardship, discrimination, social isolation, and language barriers. These factors may explain the lower prevalence rates observed in a German study of newly arrived Syrian refugees [17].

A 2020 systematic review of 28 qualitative and quantitative studies revealed that anxiety disorders affected 49 to 55% of refugees, while depression rates ranged from 11 to 49%. Common risk factors included a personal or family history of mental disorders and exposure to trauma. Socio-cultural and financial barriers were identified as major obstacles to accessing mental health and psychosocial support [18]. Another systematic review, encompassing 15 peer-reviewed studies and cross-sectional data from 8176 adult Syrian refugees across 10 countries, reported pooled prevalence rates of 40.9% CI: 20–44.1%) for depression and 26.6% CI: 19.30–31.8%) for anxiety [19]. More recently, a 2022 meta-analysis of nine studies involving 4873 adult Syrian refugees resettled in high-income Western countries found a pooled prevalence rate of 33% (95% CI, 27–40%) for any of the three major mental disorders—anxiety, depression or posttraumatic stress disorder (PTSD). Specifically, anxiety prevalence was 40% (95% CI, 31–50%) and depression prevalence was 31% (95% CI, 20–44%) [20].

These findings underscore the profound and enduring mental health challenges faced by refugees, particularly Syrian refugees, and highlight the urgent need for targeted interventions to address their psychological and psychosocial well-being.

Understanding the factors that influence psychological symptoms among Syrian refugees is crucial for guiding treatment and shaping policies in Jordan and Spain to support refugees’ integration, resettlement, and mental health. Identifying these factors helps design effective rehabilitation plans and informs therapists on key areas to address during therapy, setting realistic expectations. This research, which explores refugees’ experiences in Jordan and Spain, highlights how displacement affects their lives and can enhance therapeutic decision-making, reinforcing the importance of a client-centered approach throughout treatment. However, while the need for such insights is clear, existing research often falls short in providing a comprehensive understanding of how host country environments—such as socio-economic, legal, and cultural frameworks—shape these psychological outcomes.

While extensive research has been conducted on the mental health of Syrian refugees, the majority of studies are confined to single-country contexts or generalized refugee populations, leaving a significant gap in comparative analyses that systematically examine these influences. This study addresses this gap by conducting a cross-national comparison of Syrian refugees in Jordan and Spain, two countries that represent distinct socio-economic and policy contexts (developing vs. developed nations). By analyzing the differential impacts of these environments on stress, depression, and anxiety levels, this research provides critical insights into the mechanisms through which host country conditions shape refugee mental health. These findings not only advance academic understanding but also have direct implications for therapeutic practices and policy-making.

The contribution of this study lies in its nuanced, evidence-based understanding of how varying host country infrastructures and integration policies affect mental health outcomes. By juxtaposing Jordan, a resource-constrained setting with limited integration opportunities, against Spain, a high-resource context with more robust support systems, this research identifies actionable disparities that can inform targeted policy and intervention strategies. Ultimately, the findings aim to advance global mental health support for refugees, particularly in under-resourced settings, by offering tailored recommendations grounded in empirical evidence. This study thus bridges the gap between theoretical knowledge and practical application, ensuring that therapeutic interventions and policies are informed by a deeper understanding of the contextual factors that influence refugees’ psychological well-being.

## 2. Materials and Methods

### 2.1. Study Design and Sampling

This study employed a cross-sectional correlational design to evaluate and compare psychological symptoms among refugees in Jordan and Spain, offering critical insights to inform the planning and allocation of mental health care resources. The participants consisted of Syrian nationals aged 18 and older who had confirmed their refugee status for the past ten years. By focusing on this population, this study aimed to shed light on the mental health challenges faced by long-term refugees and provide evidence-based recommendations for targeted interventions in host countries.

Jordan and Spain were chosen as study sites due to their contrasting contexts in hosting Syrian refugees, offering a unique opportunity to examine how different socio-economic, cultural, and policy environments influence refugee mental health and integration outcomes. Jordan hosts one of the largest Syrian refugee populations globally, with over 1.3 million registered refugees. While many reside in urban areas, a significant number live in refugee camps such as Zaatari and Azraq. Jordan’s resource-constrained setting and restrictive policies create challenges in access to education, employment, and healthcare, making it an ideal context to study the mental health and socio-economic challenges faced by refugees in a low-resource environment.

In contrast, Spain represents a high-resource context with a structured integration process. Syrian refugees in Spain are dispersed across urban areas and supported by governmental and non-governmental organizations, with no formal refugee camps except in Ceuta. Spain’s approach emphasizes integration through housing, language training, and employment assistance, providing insights into refugee outcomes in a developed country with robust support systems. By comparing these two countries, this study captures the diverse realities of Syrian refugees in different host environments, enhancing the generalizability of findings and informing context-specific interventions to support refugee well-being.

This study employed a purposive sampling strategy to ensure representation of Syrian refugees in both Jordan and Spain. Participants were selected based on specific criteria, including length of displacement and legal status, to capture a diverse range of experiences. In Jordan, participants were recruited from both urban areas and refugee camps (e.g., Zaatari and Azraq), while in Spain, refugees were sampled from urban centers, major cities, smaller towns, and rural villages. This approach ensures that the sample reflects the heterogeneity of the refugee experience in both contexts.

A sample size of 200 (100 participants from each country) was determined to be sufficient for this study based on statistical power calculations and comparable studies in refugee mental health research. This sample size allowed for robust comparative analyses between the two countries while maintaining feasibility given the challenges of accessing refugee populations. While larger samples could provide additional granularity, 200 participants struck a balance between statistical reliability and practical constraints.

### 2.2. Data Collection

Participants were recruited from various settings in Jordan and Spain, including refugee camps, villages, residencies, centers, and societies, using a multi-faceted approach to ensure a diverse and representative sample. The recruitment process began by posting advertisements in social media groups specifically related to Syrian refugees in Jordan and Spain. These groups were selected for their relevance and accessibility to the target population. Additionally, we collaborated with non-governmental organizations (NGOs) working with refugees to identify potential participants.

To further expand the sample, we employed a snowball sampling technique, where initially recruited participants were asked to recommend others who met the study criteria. This approach helped us reach individuals who might not have been accessible through social media or NGOs. Throughout the recruitment process, we ensured that all participants provided informed consent before participating, and we respected their autonomy by allowing them to nominate others only if they were comfortable doing so.

This strategy enabled us to gather a diverse sample of Syrian refugees residing in various settings, including urban centers, smaller towns, and rural areas, while maintaining ethical standards and participant confidentiality. Data collection took place from February 2023 to February 2024. Each participant spent approximately 45 min completing all self-reported questionnaires in a single session. To ensure clarity and accessibility, all questions and instructions were provided in Arabic, the native language of the participants.

The questionnaires were administered in two formats: electronically and on paper. For the electronic questionnaires, all questions were mandatory, ensuring that participants could not submit their responses unless every question was answered. This approach guaranteed that we only received fully completed questionnaires. For paper-based questionnaires, which were distributed in refugee camps and centers, the researcher personally ensured that all questions were filled out before collecting the forms. This dual approach ensured data completeness and accuracy across all participants.

### 2.3. Demographical Characteristics

A demographic form was developed to collect essential information, confirm participant eligibility, and gather personal details such as age, sex, marital status, living arrangements, nationality, educational level, employment status, perceived level of discrimination, and monthly family income. This form served as a foundational tool to ensure the inclusion of eligible participants and to capture key sociodemographic variables that could influence this study’s outcomes.

### 2.4. Depression Anxiety Stress Scale (DASS-21)

Depression, Anxiety, and Stress Scale is a self-reported questionnaire developed by Lovibond and Lovibond [21] to measure negative psychological states, including depression, anxiety, and stress. It is widely used in both clinical and non-clinical settings, serving as a diagnostic aid, a mental health screener, and a tool for monitoring treatment outcomes [22,23]. The DASS-21 is a shorter version of the original 42-item DASS, with each of its three subscales “Depression”, “Anxiety”, and “Stress” containing 7 items. Each item is rated on a 4-Likert scale ranging from 0 to 3 (0: does not apply to me at all, 1: applies to me to some extent or some of the time, 2: applies to me to a great extent or a large part of the time, and 3: applies to me a lot or most of the time). For each subscale, the total score ranges from “0 to 21”, with higher scores indicating greater severity of symptoms. Scores are categorized as follows: Depression: (0–9: Normal, 10–13: Mild, 14–20: Moderate, and 21+: Severe to Extremely Severe), Anxiety: (0–7: Normal, 8–9: Mild, 10–14: Moderate, and 15+: Severe to Extremely Severe), and Stress: (0–14: Normal,15–18: Mild, 19–25: Moderate, and 26+: Severe to Extremely Severe).

Individuals whose scores fell within normal range were classified as having typical psychological well-being, showing little to no signs of stress, anxiety, or depression. These results in our study imply that certain participants were managing their situations well, even in the face of difficulties related to displacement and resettlement. Because the DASS-21 is a shortened version, each subscale score is multiplied by 2 to align with the scoring of the original 42-item DASS. The total score is presented as the sum of the three subscale scores, and percentiles are calculated based on the population sample [24]. The subscales have demonstrated high internal consistency, with Cronbach’s alpha coefficients of 0.88 for Depression, 0.82 for Anxiety, and 0.90 for Stress [22,23]. These robust psychometric properties make the DASS-21 a reliable tool for researchers and clinicians to assess current mental health conditions, monitor changes over time (e.g., during treatment), and evaluate treatment response [23].

In this study, the DASS-21 was administered in its Arabic version [25], ensuring accessibility and clarity for participants. The scale was self-reported by participants and scored by the researcher.

### 2.5. Ethical Considerations

Ethical approval for this study was secured from the Research Ethics Committee affiliated with the University of Salamanca in Spain. Participation was entirely voluntary, and individuals were free to withdraw at any time without consequence. Prior to enrollment, participants received a comprehensive explanation of the study’s objectives, potential risks, and researcher contact information. Written informed consent was obtained from all participants, ensuring they were fully aware of their rights and this study’s scope. To protect confidentiality, personal information was anonymized, and each participant was assigned a unique ID number. While this study did not involve any treatment or direct health risks, some questions had the potential to evoke negative emotions or psychological discomfort. To address this, psychological support was made available, and all participant inquiries were promptly and thoroughly addressed to ensure their well-being throughout the research process.

### 2.6. Data Analysis

Statistical analyses were conducted using the Statistical Package for the Social Sciences (SPSS) version 25.0. Categorical variables, including demographic characteristics and levels of stress, anxiety, and depression, were described using frequencies and valid percentages, expressed as numbers and percentages. Group comparisons (Jordan, Spain, and Total) were performed using the Chi-square test. The normality assumption was assessed using histograms, the Shapiro–Wilk test, and the Kolmogorov–Smirnov test. As the data were not normally distributed, Mann–Whiteny U and Kruskal–Wallis tests were used to compare continuous variables between groups. A *p*-value of less than 0.05 (α = 0.05) was considered statistically significant for all analyses.

## 3. Results

A total of 200 Syrian adults aged 18 years and above participated in the study, with 100 residing in Jordan and 100 in Spain. Table 1 summarizes the socio-demographic characteristics of the participants.

### 3.1. Stress, Anxiety, and Depression Levels

Table 2 presents the overall levels of stress, anxiety, and depression among participants. Normal levels of stress, anxiety, and depression were reported by 44.5%, 36.5%, and 31.5% of participants, respectively. However, extremely severe anxiety and moderate to severe depression were significantly higher in Jordan (66.7%, 60.4%, and 63.2%, respectively) compared with Spain (*p* = 0.022 and *p* = 0.040, respectively).

### 3.2. Sociodemographic Factors and Mental Health

Table 3 shows stress, anxiety, and depression levels by sociodemographic characteristics, irrespective of country. Clinical stress was significantly higher among females (25.5%, *p* = 0.048) and individuals aged 18–29 (16%, *p* = 0.023). Clinical anxiety was more prevalent among unemployed individuals (44.5%, *p* = 0.015), those reporting high discrimination (38.5%, *p* = 0.028), and low-income groups (39.5%, *p* = 0.016). Similarly, clinical depression was higher among unemployed individuals (44%, *p* = 0.015), those exposed to high discrimination (38%, *p* = 0.024), and low-income groups (39.5%, *p* = 0.016).

### 3.3. Cross-Country Comparisons

Table 4 compares mental health levels by sociodemographic characteristics across Jordan and Spain. In Jordan, individuals aged 18–29 exhibited significantly higher clinical stress (36%, *p* = 0.013), anxiety (68%, *p* = 0.030), and depression (60%, *p* = 0.000). Low-income families in Jordan also reported higher clinical depression (74.6%, *p* = 0.035). In Spain, clinical stress (48.4%, *p* = 0.029) and anxiety (61.3%, *p* = 0.009) were significantly higher among females. Unemployment (55.8%, *p* = 0.026) and low discrimination exposure (58%, *p* = 0.011) were also associated with clinical anxiety in Spain.

### 3.4. Statistical Analyses

Table 5 presents differences in mental health scores by sociodemographic factors, independent of country. A Kruskal–Wallis test revealed significant differences in stress (H(4) = 12.142, *p* = 0.016) and depression (H(4) = 13.154, *p* = 0.011) across age groups. Pairwise comparisons indicated higher depression among individuals aged 40–49 compared with those aged 50–59 (*p* = 0.024). A Mann–Whitney U test showed significantly higher stress among females (n = 105, mean rank = 109.34) compared with males (n = 95, mean rank = 90.73; U = 4059.500, z = −2.276, *p* = 0.023) and higher depression among females (n = 105, mean rank = 109.19) compared with males (n = 95, mean rank = 90.90; U = 4075.500, z = −2.236, *p* = 0.025).

Stress, anxiety, and depression levels also varied significantly by employment status (H(3) = 12.309, *p* = 0.006; H(3) = 11.461, *p* = 0.009; H(3) = 16.531, *p* = 0.001, respectively). Pairwise comparisons revealed significantly higher depression among unemployed individuals compared with those with full-time employment (*p* = 0.003). Income level was significantly associated with stress, anxiety, and depression (H(2) = 9.800, *p* = 0.007; H(2) = 8.033, *p* = 0.018; H(2) = 12.816, *p* = 0.002, respectively), with pairwise comparisons showing higher stress (*p* = 0.014), anxiety (*p* = 0.027), and depression (*p* = 0.012) among low-income groups compared with high-income groups.

Living arrangements also played a significant role in mental health outcomes. Individuals residing in low-social-connectivity communities and smaller villages or rural municipalities reported higher anxiety (H(4) = 10.775, *p* = 0.029) compared with other settings. Additionally, depression levels varied significantly by discrimination level (H(2) = 7.160, *p* = 0.028), with higher depression observed among individuals experiencing high discrimination compared with medium discrimination (*p* = 0.035). These findings highlight the influence of age, gender, employment, income, discrimination, and living arrangements on mental health, emphasizing the need for targeted interventions to address disparities among vulnerable groups.

Table 6 highlights country-specific differences. In Jordan, a Kruskal–Wallis test revealed significant differences in stress (H(4) = 25.056, *p* < 0.001), anxiety (H(4) = 13.398, *p* = 0.009), and depression (H(4) = 24.502, *p* < 0.001) across age groups. Pairwise comparisons showed higher stress, anxiety, and depression among individuals aged 40–49 compared with those aged 50–59 (*p* = 0.001, *p* = 0.013, and *p* = 0.002, respectively) and higher stress and depression among those aged 40–49 compared with 18–29 (*p* = 0.001 for both). Stress also varied significantly by education level (H(6) = 12.606, *p* = 0.050), while depression was significantly associated with employment status (H(3) = 10.361, *p* = 0.016) and income level (H(1) = 5.829, *p* = 0.016).

In Spain, females reported significantly higher stress (n = 62, mean rank = 54.98) compared with males (n = 38, mean rank = 43.18; U = 900.000, z = −1.985, *p* = 0.047) and higher anxiety (n = 62, mean rank = 55.49) compared with males (n = 38, mean rank = 42.36; U = 868.500, z = −2.223, *p* = 0.026). Anxiety and depression levels varied significantly by employment status (H(3) = 8.589, *p* = 0.035; H(3) = 8.595, *p* = 0.035, respectively) and discrimination level (H(2) = 8.427, *p* = 0.015; H(2) = 6.994, *p* = 0.030, respectively). Pairwise comparisons indicated higher anxiety among individuals with medium discrimination compared with low discrimination (*p* = 0.020). Stress and depression were also significantly associated with income level (H(2) = 7.818, *p* = 0.020; H(2) = 6.815, *p* = 0.033, respectively), with higher levels observed among low-income groups (*p* = 0.030 and *p* = 0.046, respectively).

These results underscore the importance of considering country-specific sociodemographic factors, such as age, gender, education, employment status, income, and discrimination level, when addressing mental health disparities and designing targeted interventions. The findings highlight the need for tailored approaches to support vulnerable refugee populations in different host country contexts.

## 4. Discussion

This study highlights the significant socio-economic and mental health challenges faced by Syrian refugees in Jordan and Spain. Key findings reveal that the majority of participants had secondary education levels, were unemployed, and had low incomes. Additionally, high rates of psychological distress were observed, with 55.5% experiencing mild to extremely severe stress, 63.5% reporting mild to extremely severe anxiety, and 68.5% exhibiting mild to extremely severe depression. These findings underscore the profound impact of displacement, economic hardship, and limited access to education and employment on the mental health of Syrian refugees.

The Syrian conflict has caused widespread displacement, disrupting the educational system and exacerbating pre-existing inequalities. Before the crisis, Syria faced educational disparities linked to parental and regional backgrounds, with increasing dropout rates among older adolescents [26]. The conflict further destabilized the education system, which had previously achieved high enrollment rates at primary and secondary levels [26]. Syrian refugees in neighboring countries, such as Lebanon, have encountered significant barriers to accessing education, despite its importance to families [27].

Our findings align with previous research indicating that Syrian refugees face substantial challenges in education, employment, and income across host countries. In Turkey, only 38.6% of refugees were employed, often in unpaid or irregular work [28]. Similarly, in Jordan, less than a fifth of refugees were employed, primarily in informal and illegal sectors [29]. In the Netherlands, Syrian refugees often combine volunteering with language lessons but rarely engage in formal education or employment [30]. Low enrollment rates in Jordan further highlight the persistent educational barriers faced by refugees [29]. Factors such as gender, age, language proficiency, and education level significantly influence employment prospects [28,30].

In Spain, refugees face vulnerable conditions, including economic uncertainty, housing poverty, and job insecurity during their first five years of integration [31]. The Spanish refugee reception system, which relies heavily on NGOs, has been criticized for its inability to effectively address these challenges [32]. Media coverage of the crisis has often focused on visual representations of the conflict, reflecting broader societal perceptions [33].

This study found elevated levels of stress, anxiety, and depression among Syrian refugees, with significant variations based on demographic and socio-economic factors. Stress was notably higher among young adults (18–29), middle-aged adults (40–49), females, the unemployed, and those with low incomes, regardless of host country. Anxiety was more prevalent among individuals aged 40–49, the unemployed, those with low social support, low-income individuals, and those facing high levels of discrimination. Similarly, depression was higher among females, individuals aged 40–49, the unemployed, those with low social support, low-income individuals, and those experiencing high discrimination.

These findings are consistent with previous research. A systematic review of adult Syrian refugees across 10 countries reported prevalence rates of 40.9% for depression and 26.6% for anxiety [19]. In Turkey, 36.1% of Syrian refugees exhibited anxiety symptoms, and 34.7% had depression symptoms [34]. Other studies found that 37.4% of refugees in camps and 47.7% in urban areas experienced probable depression [35,36]. However, a meta-analysis on conflict-affected populations reported lower rates of anxiety (21.7%) and depression (10.8%) [37].

In Jordan, Syrian refugees reported higher levels of depression and anxiety compared with those in Spain. Stress was significantly higher among young adults (18–29), middle-aged adults (40–49), married or divorced individuals, females, and those with low education. Anxiety was also associated with young and middle-aged adults. Depression was more prevalent among young adults, middle-aged individuals, females, low-income groups, the unemployed, and those with low educational levels. Previous studies in Jordan found that 26% of Syrian refugees met the criteria for depression, with restricted access to medication, chronic diseases, and limited household income being significant contributing factors [38]. Low educational levels were also linked to increased stress and depression in our study.

In Spain, lower levels of discrimination were associated with reduced depression and anxiety. Stress and depression were significantly higher among females, individuals in large families, and those with low incomes. Anxiety and depression was elevated among the unemployed, females, those at a low-income level, and those facing moderate discrimination. Depression was notably higher among individuals in large families and those with low incomes. Previous research in developed host countries, such as Norway and Germany, has highlighted the strong link between perceived discrimination and poor mental health outcomes, including depression, anxiety, and PTSD symptoms [39]. However, factors such as higher educational levels and a sense of efficacy derived from Syrian identity may buffer against the negative effects of discrimination [40].

Being female was consistently associated with higher rates of depression and anxiety, a finding supported by previous studies [34,35,36]. This may be attributed to biological factors, gender-based violence, and post-displacement challenges, such as early marriages, family restrictions, or abuse [41,42]. Changes in gender roles, such as females becoming the primary income earners due to the absence or disability of male family members, may also contribute to higher psychological distress [35].

Age was another significant factor, with refugees aged 18–29 and 40–49 showing higher levels of mental disorders. Young adults face challenges related to identity development and future planning, while middle-aged individuals often experience midlife crises and sudden life changes. However, the literature presents mixed findings, with some studies reporting higher mental illness rates among older refugees [16] and others finding younger age groups more vulnerable due to their involvement in combat or insurgency [43].

This study underscores the complex interplay of socio-economic and psychological factors affecting Syrian refugees in Jordan and Spain. The findings highlight the urgent need for targeted interventions to address mental health challenges, improve access to education and employment, and reduce discrimination. By understanding the unique challenges faced by different demographic groups, policy-makers and practitioners can develop more effective strategies to support refugee integration and well-being.

## 5. Conclusions

This study offers critical insights into the long-term psychological impact of the Syrian conflict on refugees, even years after their displacement and resettlement in host countries. It underscores the necessity of a holistic healthcare approach that addresses the complex interplay of psychological symptoms experienced both pre- and post-displacement. A deep understanding of the pre-crisis Syrian education system, coupled with the educational challenges refugees face in host countries, is essential for designing effective interventions and policies that support their integration and learning.

There is an urgent need for comprehensive mental health support that not only addresses the trauma experienced before and during displacement but also tackles the challenges of resettlement. Early screening and targeted mental health interventions can significantly improve outcomes for this vulnerable population. The findings highlight the importance of tailored support that considers the unique psychological, social, and economic challenges refugees encounter.

This study also emphasizes the critical role of combating discrimination and promoting social inclusion in enhancing mental health outcomes for Syrian refugees. It recommends the implementation of anti-discrimination policies, initiatives to foster community inclusion, and the provision of immediate psychological support. Furthermore, intensive psychological and psychiatric interventions, along with rehabilitation programs, are vital to help refugees adapt to their new environments. These efforts are essential for empowering refugees to cope effectively with their new lives and for promoting their long-term mental health and well-being.

Additionally, this study highlights the mental health benefits of regular physical activity, which has been shown to significantly alleviate symptoms of depression and anxiety. Physical activity not only provides long-term mental health benefits but also offers short-term mood improvements by reducing stress hormones, enhancing sleep quality, and triggering the release of endorphins [44]. Further research into the effects of physical activity on the mental health of refugee populations is strongly encouraged, as it could offer a valuable, accessible, and cost-effective intervention to support their psychological well-being.

In conclusion, this study calls for a multifaceted approach to addressing the mental health needs of Syrian refugees, combining early intervention, tailored support, anti-discrimination measures, community inclusion, and the promotion of physical activity to foster resilience and improve overall quality of life.

## Figures and Tables

**Table 1:** The sociodemographic and relevant characteristics of the participants.

Variables	Jordan (N = 100)		Spain (N = 100)		*p*-Value	Total (N = 200)	
	n	%	n	%		n	%
Age							
18–29	50	58.1	36	41.9	0.260	86	43
30–39	28	48.3	30	51.7		58	29
40–49	16	40	24	60		40	20
50–59	4	33.3	8	66.7		12	6
≥60	2	50	2	50		4	2
Sex							
Male	57	60	38	40	0.007	95	47.5
Female	43	41	62	59		105	52.5
Marital Status							
Married	36	35	67	65	0.000	103	51.5
Single	55	69.6	24	30.4		79	39.5
Widow	5	62.5	3	37.5		8	4
Divorced	4	40	6	60		10	5
Living Arrangement							
Big cities	35	29.2	85	70.8	0.000	120	60
Large towns	10	50	10	50		20	10
Smaller villages and rural	15	78.9	4	21.1		19	9.5
Low social support	0	0	1	100		1	.5
Camps	40	100	0	0		40	20
Educational level							
Illiterate	2	66.7	1	33.3	0.653	3	1.5
Elementary	17	40.5	25	59.5		42	21
Secondary	45	48.4	48	51.6		93	46.5
Diploma	13	56.5	10	43.5		23	11.5
Bachelors	18	56.3	14	43.8		32	16
Masters	3	75	1	25		4	2
Doctoral	2	66.7	1	33.3		3	1.5
Employment Status							
Not employed	63	45	77	55	0.013	140	70
Part-time employment	26	74.3	9	25.7		35	17.5
Full-time employment	9	40.9	13	59.1		22	11
Retired	2	66.7	1	33.3		3	1.5
Discrimination							
Low	69	50	69	50	0.750	138	69
Medium	26	48.1	28	51.9		54	27
High	5	62.5	3	37.5		8	4
Monthly Family Income							
Low income	63	52.1	58	47.9	0.121	121	60.5
Middle income	37	49.3	38	50.7		75	37.5
High income	0	0	4	100		4	2

**Table 2:** Stress, anxiety, and depression levels among adult Syrian refugees in general and according to residency.

Variables	Jordan (N = 100)		Spain (N = 100)		*p*-Value	Total (N = 200)	
	n	%	n	%		n	%
Stress, Anxiety, and Depression *							
Stress							
Normal	42	47.2	47	52.8	0.921	89	44.5
Mild	14	51.9	13	48.1		27	13.5
Moderate	15	51.7	14	48.3		29	14.5
Severe	17	56.7	13	43.3		30	15
Extremely Severe	12	48.0	13	52.0		25	12.5
Anxiety							
Normal	27	37.0	46	63.0	0.022	73	36.5
Mild	4	57.1	3	42.9		7	3.5
Moderate	19	48.7	20	51.3		39	19.5
Severe	12	50.0	12	50.0		24	12
Extremely Severe	38	66.7	19	33.3		57	28.5
Depression							
Normal	22	34.9	41	65.1	0.040	63	31.5
Mild	11	61.1	7	38.9		18	9
Moderate	29	60.4	19	39.6		48	24
Severe	12	63.2	7	36.8		19	9.5
Extremely Severe	26	50.0	26	50.0		52	26

* Depression Anxiety Stress Scale (DASS-21).

**Table 3:** Stress, anxiety, and depression according to the sociodemographic, and relevant characteristics of the participants.

Variables	Total (N = 200)														
	Stress *				*p*-Value	Anxiety *				*p*-Value	Depression *				*p*-Value
	Clinical		Not Clinical			Clinical		Not Clinical			Clinical		Not Clinical		
	n	%	n	%		n	%	n	%		n	%	n	%	
Age															
18–29	32	16.0	54	27.0	0.023	49	24.5	37	18.5	0.064	48	24.0	38	19.0	0.073
30–39	27	13.5	31	15.5		40	20.0	18	9.0		38	19.0	20	10.0	
40–49	23	11.5	17	8.5		25	12.5	15	7.5		28	14.0	12	6.0	
50–59	1	0.5	11	5.5		6	3.0	6	3.0		4	2.0	8	4.0	
≥60	1	0.5	3	1.5		0	0.0	4	2.0		1	0.5	3	1.5	
Sex															
Male	33	16.5	62	31.0	0.048	51	25.5	44	22.0	0.083	52	26.0	43	21.5	0.192
Female	51	25.5	54	27.0		69	34.5	36	18.0		67	33.5	38	19.0	
Employment status															
Not employed	63	31.5	77	38.5	0.199	89	44.5	51	25.5	0.013	88	44.0	52	26.0	0.015
Part-time	15	7.5	20	10.0		23	11.5	12	6.0		23	11.5	12	6.0	
Full-time	6	3.0	16	8.0		8	4.0	14	7.0		8	4.0	14	7.0	
Retired	0	0.0	3	1.5		0	0.0	3	1.5		0	0.0	3	1.5	
Discrimination															
Low	55	27.5	83	41.5	0.558	77	38.5	61	30.5	0.028	76	38.0	62	31.0	0.024
Medium	26	13.0	28	14.0		40	20.0	14	7.0		40	20.0	14	7.0	
High	3	1.5	5	2.5		3	1.5	5	2.5		3	1.5	5	2.5	
Monthly Family Income															
Low	55	27.5	66	33.0	0.147	79	39.5	42	21.0	0.016	79	39.5	42	21.0	0.013
Middle	29	14.5	46	23.0		41	20.5	34	17.0		40	20.0	35	17.5	
High	0	0.0	4	2.0		0	0.0	4	2.0		0	0.0	4	2.0	

* The classification of participants into clinical and non-clinical groups was based on the severity of their symptoms as measured by the Depression, Anxiety, and Stress Scale (DASS). Participants scoring in the “normal” or “mild” ranges were categorized as non-clinical, indicating the absence or sub-threshold presence of significant psychological distress. Conversely, those scoring in the “moderate”, “severe”, or “extremely severe” ranges were classified as clinical, reflecting symptom levels that meet or exceed thresholds for clinically significant mental health concerns.

**Table 4:** Levels of stress, anxiety, and depression according to the sociodemographic and relevant characteristics of the participants based on their country of residence.

Variables	Jordan															Spain														
	Stress *				*p*-Value	Anxiety *				*p*-Value	Depression *				*p*-Value	Stress *				*p*-Value	Anxiety *				*p*-Value	Depression *				*p*-Value
	Clinical		Not Clinical			Clinical		Not Clinical			Clinical		Not Clinical			Clinical		Not Clinical			Clinical		Not Clinical			Clinical		Not Clinical		
	n	%	n	%		n	%	n	%		n	%	n	%		n	%	n	%		n	%	n	%		n	%	n	%	
Age																														
18–29	18	36	32	64	0.013	34	68	16	32	0.030	30	60	20	40	0.000	14	38.9	22	61.1	0.541	15	41.7	21	58.3	0.138	18	50	18	50	0.985
30–39	14	50	14	50		20	71.4	8	28.6		21	75	7	25		13	43.3	17	56.7		20	66.7	10	33.3		17	56.7	13	43.3	
40–49	12	75	4	25		14	87.5	2	12.5		16	100	0	0		11	45.8	13	54.2		11	45.8	13	54.2		12	50	12	50	
50–59	0	0	4	100		1	25	3	75		0	0	4	100		1	12.5	7	87.5		5	62.5	3	37.5		4	50	4	50	
≥60	0	0	2	100		0	0	2	100		0	0	2	100		1	50	1	50		0	0	2	100		1	50	1	50	
Sex																														
Male	23	40.4	34	59.6	0.397	38	66.7	19	33.3	0.561	36	63.2	21	36.8	0.347	10	26.3	28	73.7	0.029	13	34.2	25	65.8	0.009	16	42.1	22	57.9	0.121
Female	21	48.8	22	51.2		31	72.1	12	27.9		31	72.1	12	27.9		30	48.4	32	51.6		38	61.3	24	38.7		36	58.1	26	41.9	
Employment status																														
None	31	49.2	32	50.8	0.391	46	73	17	27	0.164	44	69.8	19	30.2	0.181	32	41.6	45	58.4	0.361	43	55.8	34	44.2	0.026	44	57.1	33	42.9	0.098
Part-time	10	38.5	16	61.5		17	65.4	9	34.6		18	69.2	8	30.8		5	55.6	4	44.4		6	66.7	3	33.3		5	55.6	4	44.4	
Full-time	3	33.3	6	66.7		6	66.7	3	33.3		5	55.6	4	44.4		3	23.1	10	76.9		2	15.4	11	84.6		3	23.1	10	76.9	
Retired	0	0	2	100		0	0	2	100		0	0	2	100		0	0	1	100		0	0	1	100		0	0	1	100	
Discrimination																														
Low	31	44.9	38	55.1	0.958	48	69.6	21	30.4	0.337	46	66.7	23	33.3	0.352	24	34.8	45	65.2	0.225	29	42	40	58	0.011	30	43.5	39	56.5	0.015
Medium	11	42.3	15	57.7		19	73.1	7	26.9		19	73.1	7	26.9		15	53.6	13	46.4		21	75	7	25		21	75	7	25	
High	2	40	3	60		2	40	3	60		2	40	3	60		1	33.3	2	66.7		1	33.3	2	66.7		1	33.3	2	66.7	
Monthly Family Income																														
Low	30	47.6	33	52.4	0.341	47	74.6	16	25.4	0.114	47	74.6	16	25.4	0.035	25	43.1	33	56.9	0.234	32	55.2	26	44.8	0.101	32	55.2	26	44.8	0.102
Middle	14	37.8	23	62.2		22	59.5	15	40.5		20	54.1	17	45.9		15	39.5	23	60.5		19	50	19	50		20	52.6	18	47.4	
High	0	0	0	0		0	0	0	0		0	0	0	0		0	0.	4	100		0	0	4	100		0	0	4	100	

* The classification of participants into clinical and non-clinical groups was based on the severity of their symptoms as measured by the Depression, Anxiety, and Stress Scale (DASS). Participants scoring in the “normal” or “mild” ranges were categorized as non-clinical, indicating the absence or sub-threshold presence of significant psychological distress. Conversely, those scoring in the “moderate”, “severe”, or “extremely severe” ranges were classified as clinical, reflecting symptom levels that meet or exceed thresholds for clinically significant mental health concerns.

**Table 5:** The differences between the participants’ sociodemographics and relevant characteristics based on their stress, anxiety, and depression levels.

Variable		Total (N = 200)								
		Stress			Anxiety			Depression		
		µ	SD	*p*	µ	SD	*p*	µ	SD	*p*
Age (Y)	18–29	16.26	10.294	0.016	12.77	10.786	0.062	14.28	10.570	0.011
	30–39	19.10	11.290		15.07	11.438		19.66	13.529	
	40–49	21.75	14.947		16.05	13.926		22.50	15.766	
	50–59	10.67	6.569		8.67	8.105		11.17	9.590	
	≥60	9.00	15.449		1.50	1.915		11.00	16.773	
Sex	Male	15.62	10.842	0.023	12.02	11.122	0.077	14.80	11.927	0.025
	Female	19.58	12.477		15.07	11.952		19.43	13.811	
Marital State	Married	19.36	12.443	0.242	13.75	11.738	0.755	19.38	14.387	0.118
	Single	15.59	10.541		12.84	11.375		14.10	10.902	
	Widow	17.50	11.502		17.25	10.794		16.75	9.377	
	Divorced	17.40	14.909		15.60	14.104		20.20	14.680	
Living Arrangement	Big cities	16.68	11.585	0.179	11.93	10.821	0.029	16.07	13.210	0.096
	towns	18.70	14.294		14.40	14.791		18.30	14.353	
	village	21.89	11.518		19.05	10.464		23.05	13.239	
	Low social	42.00			42.00			48.00		
	Camps	17.65	10.991		15.00	11.422		16.65	10.921	
Employment Status	None	18.66	12.276	0.006	14.71	12.093	0.009	19.09	13.663	0.001
	Part-time	18.69	8.970		13.94	10.181		16.51	10.202	
	Full time	12.27	11.369		7.91	8.997		8.55	9.723	
	Retired	1.33	2.309		.67	1.155		2.67	2.309	
Discrimination	Low	17.30	11.987	0.173	13.22	12.098	0.078	16.81	13.645	0.028
	Medium	19.44	10.819		15.59	10.471		19.59	11.209	
	High	12.75	15.818		7.25	8.681		8.50	12.950	
Monthly Family Income	Low	19.19	12.004	0.007	14.74	11.679	0.018	19.54	13.803	0.002
	Middle	16.11	11.193		12.48	11.436		14.32	11.126	
	High	2.50	5.000		1.00	2.000		2.00	4.000	

**Table 6:** The differences in participants’ sociodemographic and relevant characteristics based on their stress, anxiety, and depression levels, categorized by their country of residence.

Variable		Jordan (N = 100)									Spain (N = 100)								
		Stress			Anxiety			Depression			Stress			Anxiety			Depression		
		µ	SD	*p*	µ	SD	*p*	µ	SD	*p*	µ	SD	*p*	µ	SD	*p*	µ	SD	*p*
Age (Y)	18–29	16.40	9.258	0.000	15.20	10.286	0.010	15.00	9.850	0.000	16.06	11.716	0.896	9.39	10.689	0.504	13.28	11.565	0.675
	30–39	20.71	9.966		17.36	12.172		21.00	11.757		17.60	12.378		12.93	10.462		18.40	15.090	
	40–49	29.13	10.092		23.00	13.145		29.00	9.438		16.83	15.791		11.42	12.656		18.17	17.731	
	50–59	7.50	5.745		4.00	8.000		9.00	2.582		12.25	6.714		11.00	7.559		12.25	11.732	
	≥60	2.00	2.828		1.00	1.414		4.00	0.000		16.00	22.627		2.00	2.828		18.00	25.456	
Sex	Male	17.12	9.871	0.097	14.77	11.050	0.185	16.39	10.368	0.067	13.37	11.938	0.047	7.89	10.016	0.026	12.42	13.748	0.064
	Female	21.49	11.712		18.37	12.628		21.21	12.522		18.26	12.908		12.77	10.986		18.19	14.611	
Marital State	Married	21.39	11.452	0.087	17.11	12.535	0.794	21.61	11.826	0.099	18.27	12.895	0.175	11.94	10.960	0.292	18.18	15.542	0.203
	Single	16.76	9.959		15.24	11.337		15.85	10.811		12.92	11.542		7.33	9.577		10.08	10.223	
	Widow	18.00	8.124		18.00	5.831		18.40	8.173		16.67	18.148		16.00	18.330		14.00	12.490	
	Divorced	29.50	13.503		22.00	18.690		26.00	15.748		9.33	9.771		11.33	9.688		16.33	13.938	
Living Arrangement	Big cities	19.77	9.687	0.566	16.86	12.061	0.748	18.51	11.348	0.470	15.41	12.106	0.152	9.91	9.631	0.149	15.06	13.841	0.221
	Towns	18.60	15.262		16.60	17.050		20.00	14.787		18.80	14.085		12.20	12.665		16.60	14.485	
	village	21.07	10.443		18.40	8.692		22.13	11.401		25.00	16.452		21.50	17.156		26.50	20.616	
	Low social	-	-		-	-		-	-		42.00			42.00			48.00		
	Camps	17.65	10.991		15.00	11.422		16.65	10.921		-	-		-	-		-	-	
Educational Level	Illiterate	31.00	12.728	0.050	24.00	16.971	0.347	36.00	5.657	0.068	16.00		0.413	20.00		0.482	16.00		0.475
	Elementary	14.00	8.888		13.06	9.168		15.65	11.118		17.04	12.532		11.76	10.682		19.44	14.861	
	Secondary	20.13	10.999		17.02	12.282		19.73	12.222		17.13	12.796		10.42	11.448		15.83	14.468	
	Diploma	22.92	8.509		17.38	10.751		19.54	8.048		10.80	12.831		7.20	8.284		11.20	16.006	
	Bachelors	19.11	11.827		17.89	13.590		18.00	11.167		16.86	12.978		13.86	11.190		15.71	13.786	
	Masters	4.67	5.033		2.67	3.055		2.00	2.000		32.00			12.00			6.00		
	Doctoral	19.00	7.071		20.00	8.485		18.00	5.657		0.00			.00			.00		
Employment Status	None	20.35	11.498	0.067	18.25	12.467	0.089	20.92	12.401	0.016	17.27	12.785	0.121	11.82	11.037	0.035	17.58	14.522	0.035
	Part-time	18.62	9.047		14.08	10.237		16.23	8.145		18.89	9.280		13.56	10.620		17.33	15.297	
	Full time	14.44	8.293		12.67	8.944		10.89	8.192		10.77	13.205		4.62	7.719		6.92	10.665	
	Retired	2.00	2.828		1.00	1.414		4.00	0.000		0.00			0.00			0.00		
Discrimination	Low	19.71	11.216	0.581	17.22	12.500	0.285	18.96	11.900	0.286	14.90	12.327	0.096	9.22	10.303	0.015	14.67	14.973	0.030
	Medium	18.15	8.853		15.46	10.001		18.62	9.512		20.64	12.413		15.71	11.072		20.50	12.691	
	High	13.60	15.582		8.40	9.209		10.80	15.595		11.33	19.630		5.33	9.238		4.67	8.083	
Monthly Family Income	Low	19.68	11.394	0.446	17.30	11.641	0.251	20.54	11.569	0.016	18.66	12.712	0.020	11.97	11.167	0.076	18.45	15.912	0.033
	Middle	17.84	9.937		14.65	12.120		14.92	10.725		14.42	12.189		10.37	10.458		13.74	11.617	
	High	-	-		-	-		-	-		2.50	5.000		1.00	2.000		2.00	4.000	

## Data Availability

The raw data supporting the conclusions of this article will be made available by the authors on request.

## Abbreviations

The following abbreviations are used in this manuscript:

CI	Confidence interval
DASS-21	Depression Anxiety Stress Scale
LSD	Least significant difference
PTSD	Post-traumatic stress disorder
SPSS	Statistical Package for the Social Sciences
UNHCR	United Nations High Commissioner for Refugees
